# 2-Amino­ethanaminium iodide

**DOI:** 10.1107/S160053681202065X

**Published:** 2012-05-16

**Authors:** Alan R. Kennedy, Maurice O. Okoth

**Affiliations:** a Department of Pure & Applied Chemistry, WestCHEM, University of Strathclyde, 295 Cathedral Street, Glasgow G1 1XL, Scotland; b Department of Chemistry and Biochemistry, Chepkoilel University College, PO Box 1125-30100, Eldoret, Kenya

## Abstract

The title salt, [NH_3_CH_2_CH_2_NH_2_]^+^·I^−^, has an array structure based on strong inter­molecular N—H⋯N hydrogen bonding formed between the ammonium and amine groups of adjacent cations. This inter­action gives a helical chain of cations that runs parallel to the *b* axis. The four remaining NH group H atoms all form hydrogen bonds to the iodide anion, and these iodide anions lie in channels parallel to the cation–cation chains.

## Related literature

For syntheses and structures of salt forms of the related ethyl­ene-1,2-diammonium, see: Chen (2009 ▶); Saidi *et al.* (2011 ▶). For a structural example of a complex of ethyl­ene-1,2-diammonium, see: Zhang *et al.* (2006 ▶). For the synthesis that gave the title compound as a by-product, see: Kennedy *et al.* (2011 ▶). For C–N bond length changes in another monoprotonated symmetrical diamine, see: Craig *et al.* (2012 ▶).
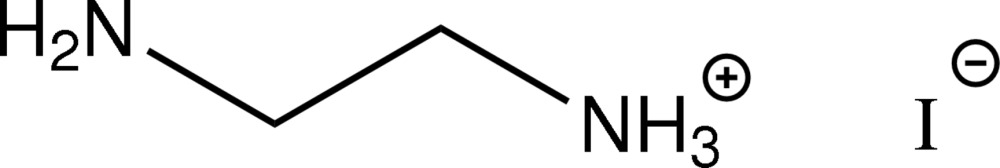


## Experimental

### 

#### Crystal data

C_2_H_9_N_2_^+^·I^−^
*M* *_r_* = 188.01
Orthorhombic, 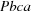

*a* = 8.1380 (2) Å
*b* = 8.6259 (2) Å
*c* = 16.7854 (6) Å
*V* = 1178.29 (6) Å^3^
*Z* = 8
Mo *K*α radiation
μ = 5.29 mm^−1^
*T* = 123 K
0.28 × 0.08 × 0.04 mm

#### Data collection

Oxford Diffraction Gemini S diffractometer
Absorption correction: multi-scan (*CrysAlis PRO*; Oxford Diffraction, 2010 ▶) *T*_min_ = 0.405, *T*_max_ = 0.805
13735 measured reflections
1675 independent reflections
1405 reflections with *I* > 2σ(*I*)
*R*_int_ = 0.025

#### Refinement

*R*[*F* ^2^ > 2σ(*F* ^2^)] = 0.016
*wR*(*F* ^2^) = 0.035
*S* = 1.08
1675 reflections
83 parameters
All H-atom parameters refined
Δρ_max_ = 0.58 e Å^−3^
Δρ_min_ = −0.39 e Å^−3^

### 

Data collection: *CrysAlis PRO* (Oxford Diffraction, 2010 ▶); cell refinement: *CrysAlis PRO*; data reduction: *CrysAlis PRO*; program(s) used to solve structure: *SIR92* (Burla *et al.*, 2005 ▶); program(s) used to refine structure: *SHELXL97* (Sheldrick, 2008 ▶); molecular graphics: *ORTEP-3* (Farrugia, 1997 ▶) and *X-SEED* (Barbour, 2001 ▶); software used to prepare material for publication: *SHELXL97*.

## Supplementary Material

Crystal structure: contains datablock(s) global, I. DOI: 10.1107/S160053681202065X/br2201sup1.cif

Structure factors: contains datablock(s) I. DOI: 10.1107/S160053681202065X/br2201Isup2.hkl

Supplementary material file. DOI: 10.1107/S160053681202065X/br2201Isup3.cml

Additional supplementary materials:  crystallographic information; 3D view; checkCIF report

## Figures and Tables

**Table 1:** Hydrogen-bond geometry (Å, °)

*D*—H⋯*A*	*D*—H	H⋯*A*	*D*⋯*A*	*D*—H⋯*A*
N1—H1*N*⋯N2^i^	1.06 (2)	1.75 (2)	2.805 (2)	173.0 (19)
N1—H2*N*⋯I1	0.882 (19)	3.231 (18)	3.6502 (14)	111.6 (13)
N1—H2*N*⋯I1^ii^	0.882 (19)	2.820 (19)	3.6047 (14)	148.9 (15)
N1—H3*N*⋯I1^iii^	0.84 (2)	2.78 (2)	3.5713 (16)	157.9 (16)
N2—H4*N*⋯I1^iv^	0.84 (2)	2.96 (2)	3.7346 (16)	155.5 (16)
N2—H5*N*⋯I1^v^	0.866 (19)	3.152 (19)	3.9328 (15)	151.2 (14)

Symmetry codes: (i) 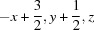; (ii) 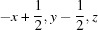; (iii) 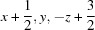; (iv) 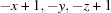; (v) 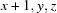.

**Fig. 1.**
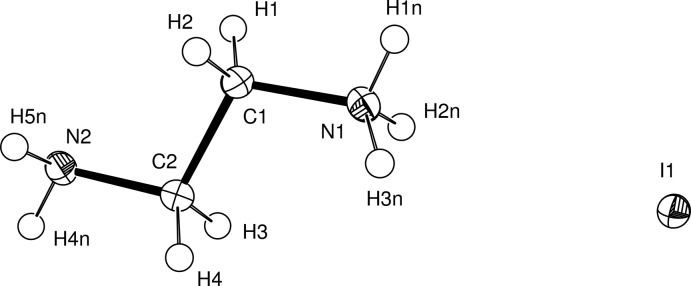
The molecular structure of (I). Displacement ellipsoids are drawn at the 50% probability level with H-atoms drawn as spheres of arbitary size.

**Fig. 2.**
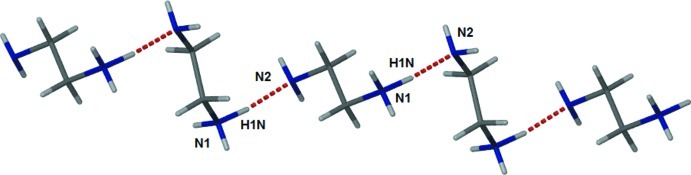
Hydrogen bonding forms helical, one-dimensional chains of cations that propagate in the crystallographic b direction.

**Fig. 3.**
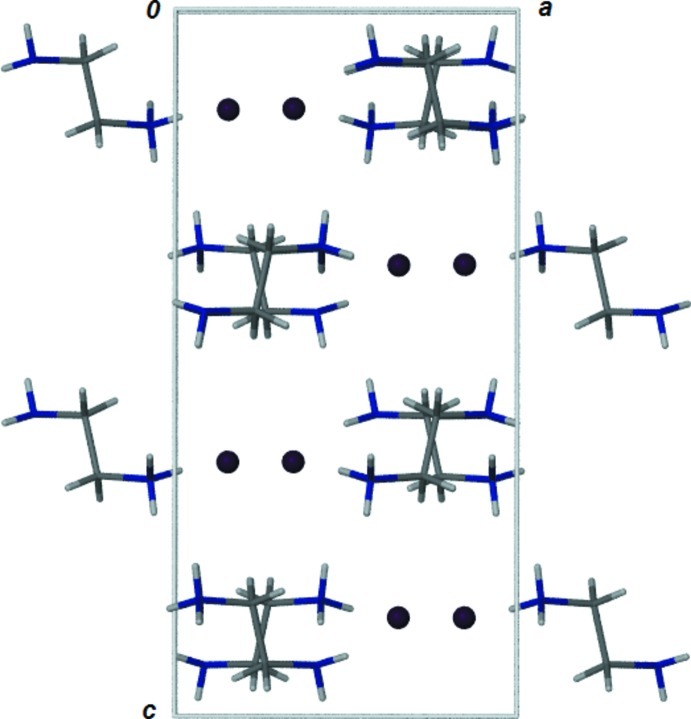
Packing in (I) viewed along the b direction.

## supplementary crystallographic information

### Comment

Despite the common use of ethylene-1,2-diamine as a ligand, there are suprisingly few metal containing crystal structures that feature its cationic form ethylene-1,2-diammonium (for an example see Zhang *et al.*, 2006) and only two structures of simple salt forms of ethylene-1,2-diammonium (Chen, 2009; Saidi *et al.*, 2011). There appears to be no previous reports of structures that contain the singley protonated cation, NH_3_CH_2_CH_2_NH_2_.

Crystals of ethylene-2-amine-1-ammonium iodide (I) were recovered whilst trying to replicate the synthesis of the macrocyclic species 5,7,7,12,14,14-hexamethyl-4,8-diaza-1,11 -diazoniocyclotetradeca-4,11-diene diiodide, the first step of which is addition of HI to ethylene-1,2-diamine in ethanol (Kennedy *et al.*, 2011). Investigation of the structure cleary showed that the base is protonated at only one site, see Figure 1. This is confirmed by location and independent refinement of the hydrogen atoms and by the slight lengthening of the C1—N1 bond as compared to the C2—N2 bond (compare 1.484 (2) and 1.467 (2) Å). Similar differences are seen in other symmetrical diamines that have been monoprotonated (see for example Craig *et al.*, 2012).

Atom H1N is a hydrogen bond donor that interacts with N2 to form the relatively short cation to cation hydrogen bond that gives the one dimensional helical chain running in the *b* direction, as shown in Figure 2. The four other N—H hydrogen atoms all interact with the iodide anion (N···I range 3.5713 (16) to 3.9328 (15) Å), see Table 1 for details. These interactions combine to give the packing motif shown in Figure 3, with channels of anions parallel to the *b* direction and thus also parallel to the cation-cation hydrogen bonded chains.

### Experimental

Crystals of (I) were obtained from ethanol solution.

### Refinement

All the H-atoms were found through difference synthesis and refined isotropically. The N1 to H1N distance is 1.06 (2) Å. This forms part of the N—H···N hydrogen bond and may reflect some small degree of positional disorder over two sites.

### Crystal data

**Table:**

C_2_H_9_N_2_^+^·I^−^	*F*(000) = 704
*M**_r_* = 188.01	*D*_x_ = 2.120 Mg m^−^^3^
Orthorhombic, *P**b**c**a*	Mo *K*α radiation, λ = 0.71073 Å
Hall symbol: -P 2ac 2ab	Cell parameters from 7738 reflections
*a* = 8.1380 (2) Å	θ = 3.4–30.4°
*b* = 8.6259 (2) Å	µ = 5.29 mm^−^^1^
*c* = 16.7854 (6) Å	*T* = 123 K
*V* = 1178.29 (6) Å^3^	Cut needle, colourless
*Z* = 8	0.28 × 0.08 × 0.04 mm

### Data collection

**Table:**

Oxford Diffraction Gemini S diffractometer	1675 independent reflections
Radiation source: fine-focus sealed tube	1405 reflections with *I* > 2σ(*I*)
Graphite monochromator	*R*_int_ = 0.025
ω scans	θ_max_ = 30.5°, θ_min_ = 3.5°
Absorption correction: multi-scan (*CrysAlis PRO*; Oxford Diffraction, 2010)	*h* = −11→11
*T*_min_ = 0.405, *T*_max_ = 0.805	*k* = −12→12
13735 measured reflections	*l* = −23→22

### Refinement

**Table:**

Refinement on *F*^2^	Secondary atom site location: difference Fourier map
Least-squares matrix: full	Hydrogen site location: difference Fourier map
*R*[*F*^2^ > 2σ(*F*^2^)] = 0.016	All H-atom parameters refined
*wR*(*F*^2^) = 0.035	*w* = 1/[σ^2^(*F*_o_^2^) + (0.0188*P*)^2^ + 0.0175*P*] where *P* = (*F*_o_^2^ + 2*F*_c_^2^)/3
*S* = 1.08	(Δ/σ)_max_ = 0.003
1675 reflections	Δρ_max_ = 0.58 e Å^−^^3^
83 parameters	Δρ_min_ = −0.39 e Å^−^^3^
0 restraints	Extinction correction: *SHELXL97* (Sheldrick, 2008), Fc^*^=kFc[1+0.001xFc^2^λ^3^/sin(2θ)]^-1/4^
Primary atom site location: structure-invariant direct methods	Extinction coefficient: 0.00618 (17)

### Special details

**Table:**

Experimental. Absorption correction: CrysAlis PRO (Oxford Diffraction, 2010). Empirical absorption correction using spherical harmonics, implemented in SCALE3 ABSPACK scaling algorithm.
Geometry. All s.u.'s (except the s.u. in the dihedral angle between two l.s. planes) are estimated using the full covariance matrix. The cell s.u.'s are taken into account individually in the estimation of s.u.'s in distances, angles and torsion angles; correlations between s.u.'s in cell parameters are only used when they are defined by crystal symmetry. An approximate (isotropic) treatment of cell s.u.'s is used for estimating s.u.'s involving l.s. planes.
Refinement. Refinement of *F*^2^ against ALL reflections. The weighted *R*-factor *wR* and goodness of fit *S* are based on *F*^2^, conventional *R*-factors *R* are based on *F*, with *F* set to zero for negative *F*^2^. The threshold expression of *F*^2^ > 2σ(*F*^2^) is used only for calculating *R*-factors(gt) *etc*. and is not relevant to the choice of reflections for refinement. *R*-factors based on *F*^2^ are statistically about twice as large as those based on *F*, and *R*- factors based on ALL data will be even larger.

### Fractional atomic coordinates and isotropic or equivalent isotropic displacement parameters (Å^2^)

**Table:**

	*x*	*y*	*z*	*U*_iso_*/*U*_eq_	
I1	0.153737 (12)	0.243717 (9)	0.639779 (7)	0.01478 (5)	
N1	0.57239 (17)	0.09977 (16)	0.66467 (10)	0.0152 (3)	
N2	0.91366 (18)	−0.12957 (16)	0.57234 (10)	0.0166 (3)	
C1	0.72638 (19)	0.00806 (18)	0.65975 (11)	0.0156 (3)	
C2	0.7645 (2)	−0.03399 (18)	0.57391 (10)	0.0164 (3)	
H1	0.720 (2)	−0.083 (2)	0.6923 (11)	0.022 (5)*	
H2	0.812 (2)	0.073 (2)	0.6768 (12)	0.025 (5)*	
H3	0.677 (2)	−0.0965 (19)	0.5534 (11)	0.020 (5)*	
H4	0.771 (2)	0.0590 (19)	0.5396 (11)	0.018 (4)*	
H1N	0.573 (3)	0.206 (3)	0.6334 (13)	0.030 (5)*	
H2N	0.487 (2)	0.042 (2)	0.6523 (12)	0.027 (5)*	
H3N	0.561 (2)	0.131 (2)	0.7119 (12)	0.028 (6)*	
H4N	0.932 (2)	−0.161 (2)	0.5259 (13)	0.029 (5)*	
H5N	0.998 (2)	−0.073 (2)	0.5848 (13)	0.029 (6)*	

### Atomic displacement parameters (Å^2^)

**Table:**

	*U*^11^	*U*^22^	*U*^33^	*U*^12^	*U*^13^	*U*^23^
I1	0.01452 (7)	0.01487 (7)	0.01494 (8)	0.00109 (3)	−0.00020 (4)	−0.00022 (4)
N1	0.0146 (7)	0.0161 (7)	0.0150 (8)	−0.0014 (5)	0.0018 (6)	−0.0016 (5)
N2	0.0151 (7)	0.0194 (7)	0.0152 (8)	0.0000 (5)	0.0019 (6)	−0.0009 (6)
C1	0.0140 (8)	0.0188 (8)	0.0141 (8)	−0.0001 (6)	−0.0006 (6)	0.0007 (6)
C2	0.0164 (8)	0.0191 (7)	0.0136 (8)	0.0010 (6)	0.0007 (7)	0.0010 (6)

### Geometric parameters (Å, º)

**Table:**

N1—C1	1.484 (2)	N2—H5N	0.866 (19)
N1—H1N	1.06 (2)	C1—C2	1.518 (2)
N1—H2N	0.882 (19)	C1—H1	0.959 (18)
N1—H3N	0.84 (2)	C1—H2	0.937 (19)
N2—C2	1.467 (2)	C2—H3	0.959 (17)
N2—H4N	0.84 (2)	C2—H4	0.989 (18)
			
C1—N1—H1N	115.5 (12)	C2—C1—H1	110.8 (11)
C1—N1—H2N	110.4 (12)	N1—C1—H2	107.0 (11)
H1N—N1—H2N	112.4 (17)	C2—C1—H2	106.3 (13)
C1—N1—H3N	108.5 (13)	H1—C1—H2	110.7 (18)
H1N—N1—H3N	100.7 (16)	N2—C2—C1	108.70 (14)
H2N—N1—H3N	108.6 (17)	N2—C2—H3	107.1 (10)
C2—N2—H4N	110.2 (13)	C1—C2—H3	108.8 (11)
C2—N2—H5N	109.6 (12)	N2—C2—H4	113.8 (10)
H4N—N2—H5N	105.2 (18)	C1—C2—H4	111.7 (11)
N1—C1—C2	110.67 (14)	H3—C2—H4	106.6 (15)
N1—C1—H1	111.2 (11)		
			
N1—C1—C2—N2	−177.75 (12)		

### Hydrogen-bond geometry (Å, º)

**Table:**

*D*—H···*A*	*D*—H	H···*A*	*D*···*A*	*D*—H···*A*
N1—H1*N*···N2^i^	1.06 (2)	1.75 (2)	2.805 (2)	173.0 (19)
N1—H2*N*···I1	0.882 (19)	3.231 (18)	3.6502 (14)	111.6 (13)
N1—H2*N*···I1^ii^	0.882 (19)	2.820 (19)	3.6047 (14)	148.9 (15)
N1—H3*N*···I1^iii^	0.84 (2)	2.78 (2)	3.5713 (16)	157.9 (16)
N2—H4*N*···I1^iv^	0.84 (2)	2.96 (2)	3.7346 (16)	155.5 (16)
N2—H5*N*···I1^v^	0.866 (19)	3.152 (19)	3.9328 (15)	151.2 (14)

Symmetry codes: (i) −*x*+3/2, *y*+1/2, *z*; (ii) −*x*+1/2, *y*−1/2, *z*; (iii) *x*+1/2, *y*, −*z*+3/2; (iv) −*x*+1, −*y*, −*z*+1; (v) *x*+1, *y*, *z*.
